# Clinically applicable GABA receptor positive allosteric modulators promote ß-cell replication

**DOI:** 10.1038/s41598-017-00515-y

**Published:** 2017-03-23

**Authors:** Jide Tian, Hoa Dang, Blake Middleton, Daniel L. Kaufman

**Affiliations:** Department of Molecular and Medical Pharmacology, University of California, Los Angeles, USA

## Abstract

A key goal of diabetes research is to develop treatments to safely promote human ß-cell replication. It has recently become appreciated that activation of γ-aminobutyric acid receptors (GABA-Rs) on ß-cells can promote their survival and replication. A number of positive allosteric modulators (PAMs) that enhance GABA’s actions on neuronal GABA_A_-Rs are in clinical use. Repurposing these GABA_A_-R PAMs to help treat diabetes is theoretically appealing because of their safety and potential to enhance the ability of GABA, secreted from ß-cells, or exogenously administered, to promote ß-cell replication and survival. Here, we show that clinically applicable GABA_A_-R PAMs can increase significantly INS-1 ß-cell replication, which is enhanced by exogenous GABA application. Furthermore, a GABA_A_-R PAM promoted human islet cell replication *in vitro*. This effect was abrogated by a GABA_A_-R antagonist. The combination of a PAM and low levels of exogenous GABA further increased human islet cell replication. These findings suggest that PAMs may potentiate the actions of GABA secreted by islet ß-cells on GABA_A_-Rs and provide a new class of drugs for diabetes treatment. Finally, our findings may explain a past clinical observation of a GABA_A_-R PAM reducing HbA1c levels in diabetic patients.

## Introduction

New therapeutic approaches are needed to safely promote ß-cell replication and increase ß-cell mass in individuals with type 1 and 2 diabetes (T1D and T2D). Rodent and human ß-cells have been long known to express the GABA synthetic enzyme glutamic acid decarboxylase (GAD), as well as GABA_A_-Rs and GABA_B_-Rs^1^. Recent studies have shown that the activation of ß-cell GABA_A_-Rs and GABA_B_-Rs can promote their survival, replication and mass^2–6^. Moreover, long-term GABA treatment can promote α-cell transdifferentiation into ß-cells by activation of GABA_A_-R^7^.

GABA_A_-R PAMs enhance the action of GABA. They do not bind to the GABA-binding site, but rather elsewhere on GABA_A_-Rs. While they do not have agonist function to open the GABA_A_-R chloride channel, they increase in Cl^−^ conductance when GABA is bound to the receptor^8^. Because GABA has little to no ability to pass through the blood brain barrier (BBB), BBB-permeable GABA_A_-R PAMs, such as the benzodiazepines, have been used to enhance the action of GABA secreted by neurons in the central nervous system (CNS) in order to treat CNS disorders such as seizures, insomnia, and anxiety. It is an open question whether these GABA_A_-R PAMs can be repurposed to increase the capacity of ß-cell-secreted GABA to promote ß-cell mitogenesis.

**Figure 1:**
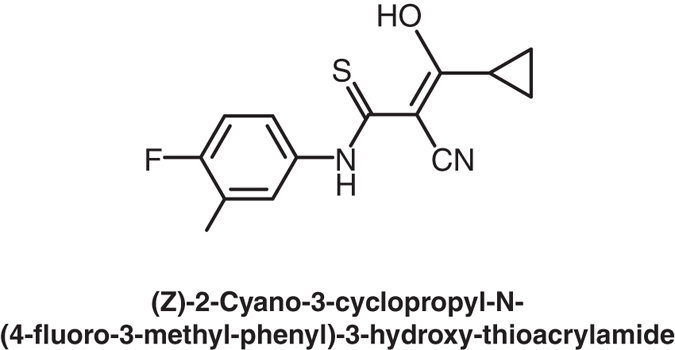
The structure of AP-3.

In the current study, we first assessed whether the rat ß-cell cell line INS-1 expressed the GABA_A_-R subunits that confer sensitivity to benzodiazepines and whether they were capable of synthesizing GABA. We then assessed the ability of clinically-applicable GABA_A_-Rs PAMs to promote the replication of rat INS-1 ß-cells. Specifically, we tested alprazolam (Xanax), midazolam, and clonazepam, which represent different classes of benzodiazepines (triazolo, imidazo, 7-nitro and 2-keto compounds, respectively). Alprazolam has been widely used for treating anxiety since the early 1980’s and nearly 50 million prescriptions are written annually for this medication. It is safe for long-term use when used as directed^9, 10^. In addition, we tested AP3, a newly developed non-benzodiazepine GABA_A_-R PAM. AP3 does not pass through the BBB and its structure is shown in Fig. 1. We then examined the effect of alprazolam, with, or without, exogenous GABA, on human islet cell replication *in vitro*. Our results using INS-1 cells and human islets indicated that treatment with a PAM alone, and to a greater extent the combination of a low dose of a PAM and exogenous GABA, significantly increased INS-1 ß-cell proliferation and human islet cell replication. This proof-of-principle study suggests that GABA_A_-R PAMs may be a new class of drugs to aid in diabetes treatment.

## Results

### INS-1 cells express benzodiazepine-binding GABA_A_-R subunits

GABA can enhance INS-1 cell proliferation^3^, but it is unknown whether INS-1 cells express the GABA_A_-subunits that are sensitive to benzodiazepines. Publically accessible profiles of INS-1 cell gene expression are inconsistent in terms of the presence or absence of various GABA_A_-R subunit transcripts, perhaps due to their low level of expression. We therefore tested INS-1 cell RNA for the presence of transcripts which encode the GABA_A_-R subunits that confer sensitivity to benzodiazepines (α_1_, α_2_, α_3_, and α_5_)^11, 12^ using quantitative real-time PCR (qPCR). We detected α_1_, α_3_, and α_5_ transcripts as well as ß1, γ1, γ2, γ3 subunits (data not shown), indicating that INS-1 cells may express GABA_A_-Rs that are sensitive to benzodiazepines.

### INS-1 cells synthesize GABA at low levels

**Figure 2:**
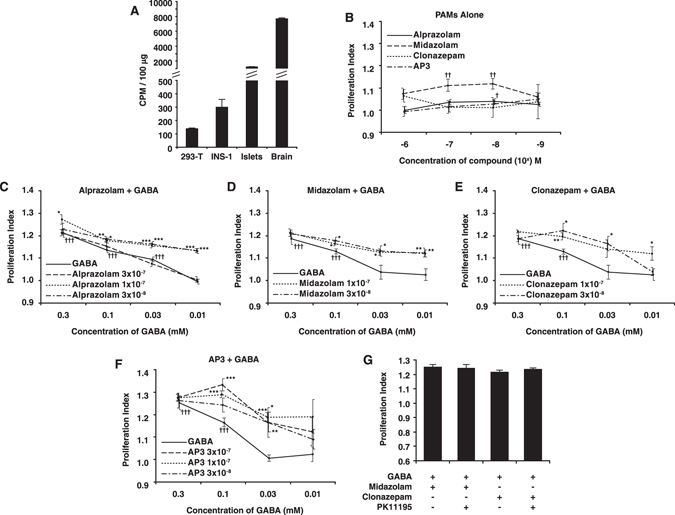
(**A**) GAD enzymatic activity in INS-1 cells. The GAD enzymatic activity within homogenates was assessed using a standard CO^2^ trapping assay as described in Methods. Data shown are mean CPM + /− SEM from a representative assay from three experiments. (**B**) Effect of PAMs on INS-1 cell proliferation. INS-1 cells were cultured with the indicated PAM at a dose range of 10^−9^ to 10^−6^ M and assessed for their proliferation. Data shown are the average rate of proliferation relative to that of cultures with media alone (designated as 1). INS-1 cells were treated with alprazolam (**C**), midazolam (**D**), clonazepam (**E**), or AP-3 (**F**) at the indicated concentrations along with a dose range of GABA. Control cultures were incubated with GABA alone at the indicated concentration (solid lines). ^††††^p < 0.001 versus control cultures with medium alone. *p < 0.05, **p < 0.01, ***p < 0.001 versus cultures with the same dose of GABA, determined by Student T test. G) INS-1 cells were cultured with GABA (0.3 mM) and midazolam or clonazepam (100 nM) with, or without, the TSPO inhibitor PK11195 (1 µM) for 48 hrs.

ß-cells express GAD and secrete GABA, which regulates islet ß-cell and α-cell function in an autocrine or paracrine fashion^1, 13^. Whether INS-1 cells also synthesize GABA is unknown. We therefore examined GAD enzymatic activity in INS-1 cells, human islets and mouse brains, as well as control 293 T cells. We found that the levels of GAD activity in INS-1 cells were considerably lower than that in human islets, which in turn were lower than that in mouse brains (Fig. 2A). Nonetheless, the levels of GAD activity in INS-1 cells were consistently about 2-fold higher than that of negative control 293 T cells, suggesting that INS-1 cells are capable of producing a low level of GABA.

### GABA_A_-Rs PAMs enhance INS-1 cell proliferation *in vitro*

Given that INS-1 cells express benzodiazepine sensitive GABA_A_-R subunits and have the capacity to synthesize GABA, we tested whether clinically applicable GABA_A_-R PAMs could enhance their proliferation. INS-1 cells were treated with, or without, different concentrations of each PAM and their proliferation was determined by ^3^H-thymidine incorporation. Treatment with alprazolam or midazolam at 10 or/and 100 nM, but not with clonazepam or AP-3, significantly stimulated proliferation of INS-1 cells (Fig. 2B). Hence, some GABA_A_-R PAMs enhanced endogenous GABA-promoted proliferation of INS-1 cells *in vitro*.

### PAMs enhance GABA-stimulated INS-1 cell proliferation

Next, we examined the impact of the combination of exogenous GABA and a PAM on the proliferation of INS-1 cells. We found that treatment with GABA (alone) at 0.03–0.3 mM increased proliferation of INS-1 cells (Fig. 2C–F), consistent with a previous report^3^. While treatment with 0.01 mM GABA did not significantly increase INS-1 proliferation (Fig. 2C–F), treatment with the same dose of GABA in the presence of 30 or 100 nM alprazolam significantly increased INS-1 cell proliferation, which was significantly higher than that of alprazolam alone or even a 10-fold higher concentration of GABA alone (0.1 mM, Fig. 2C). However, alprazolam had less ability to enhance INS-1 cell proliferation induced by a high concentration of GABA (0.1 or 0.3 mM), perhaps because high concentrations of GABA already induced maximal GABA_A_-R responses. In a similar fashion, treatment with low doses of midazolam, clonazepam, or AP-3 enhanced GABA-stimulated INS-1 cell proliferation (Fig. 2D–F). Moreover, similar patterns of PAM-enhanced exogenous GABA-stimulated INS-1 cell proliferation were detected using MTT assays (Fig. S1). Thus, GABA_A_-R PAMs can potentiate GABA-induced ß-cell proliferation *in vitro*.

### GABA_A_-R PAMs promote INS-1 cell proliferation independent of the mitochondrial translocator protein (TSPO)

Some benzodiazepines bind to the mitochondrial translocator protein (TSPO), which was previously referred to as a “peripheral benzodiazepine receptor”. Alprazolam does not bind to TSPO^14^. To test whether midazolam and clonazepam may enhance INS-1 cell proliferation through binding to the TSPO, INS-1 cells were treated with, or without, the TSPO inhibitor PK11195 and stimulated with 0.3 mM GABA and 100 nM midazolam or clonazepam for 48 h. Treatment reating prowith PK1119 did not significantly affect the ability of GABA and the tested benzodiazepines to enhance INS-1 cell replication (Fig. 2G), indicating that PAMs enhanced GABA-stimulated proliferation of INS-1 cells independent of TSPO.

### Alprazolam enhances the ability of endogenous GABA to promote human islet cell replication *in vitro*

**Figure 3:**
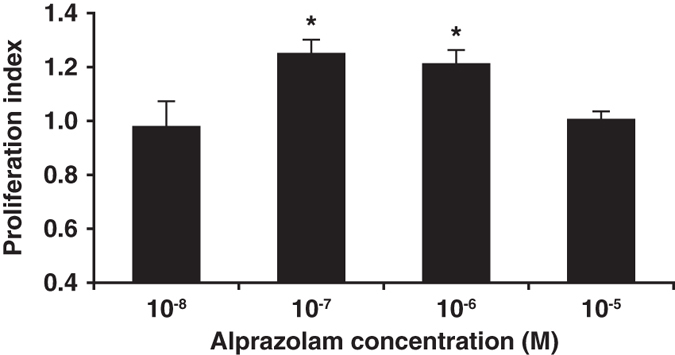
Alprazolam enhances human islet cell replication. Fresh human islets were treated in triplicate with the indicated dosage of GABA, together with, or without, the indicated PAM as described in Methods. Data shown are the average rate of proliferation relative to that of cultures with medium alone (designated as 1). N = three independent studies. *p < 0.05, determined by Student T test.

**Figure 4:**
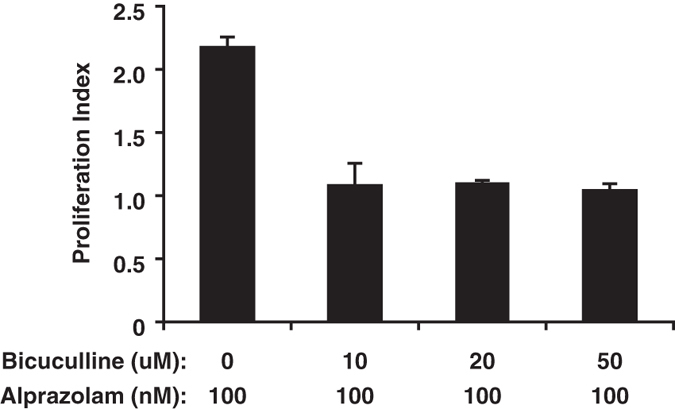
Treatment with bicuculline ablates the ability of alprazolam to enhance islet cell replication. Human islets were incubated with alprazolam (100 nM) together with a standard dose range of bicuculline (0–50 uM). Data shown are the average rate of proliferation relative to that of cultures with medium alone (designated as 1).

We next tested whether a PAM could enhance human islet cell replication. We focused on testing alprazolam because of its safety record^9, 10^ and the finding that alprazolam treatment reduced HbA1c in diabetic patients^15^. Human islets were treated with, or without, a dose range of alprazolam and the islet cell proliferation was determined by ^3^H-thymidine incorporation. We found that low concentrations of alprazolam promoted significantly human islet cell proliferation (Fig. 3). Alprazolam’s pro-mitotic effects were abrogated by the presence of the GABA_A_-R antagonist bicuculline^16^ (Fig. 4) or the Ca^2+^ channel blocker nifedipine (1 µM, data not shown). These observations are consistent with the notion that this PAM enhances GABA_A_-R-mediated activation of the PI3K/Akt signaling to enhance human islet cell proliferation^3^.

### The combination of alprazolam and GABA has enhanced ability to promote human islet cell replication at low dosage *in vitro*

**Figure 5:**
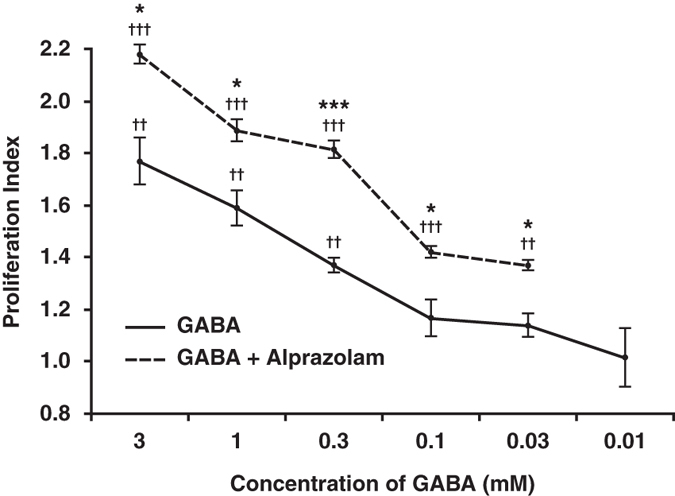
Alprazolam enhances GABA’s ability to promote human islet cell replication. Human islets were incubated with a dose range of GABA together with alprazolam (100 ng/ml) for 4 days in the presence of ^3^H thymidine. Data shown are the average rate of proliferation relative to that of cultures with medium alone (designated as 1) in a representative study. N = two independent studies with triplicate cultures. The results were very similar in both studies. ^††^p < 0.01 and ^†††^p < 0.001for GABA, or GABA + alprazolam vs. control medium alone; *p < 0.05 and ***P < 0.01 for GABA + alprazolam vs. GABA alone, determined by Student T-test.

Finally, we examined whether alprazolam could potentiate the ability of exogenous GABA to promote human islet cell replication *in vitro*. Human islets were treated with, or without, different concentrations of GABA in the presence or absence of 100 nM alprazolam. We found that GABA (alone) at 0.3-3 mM, but not a lower dose, significantly enhanced human islet cell proliferation in a dose-dependent manner (Fig. 5). Co-treatment with alprazolam and GABA significantly increased human islet cell proliferation relative to the corresponding GABA (alone) doses (Fig. 5). Notably, while treatment with 0.03 mM GABA did not significantly enhance human islet cell proliferation, treatment with the same dose of GABA together with alprazolam significantly increased human islet cell proliferation. To achieve a similar level of proliferation using GABA alone required a 10-fold higher level of GABA (0.3 mM, Fig. 5). Thus, alprazolam greatly potentiated the effect of exogenous GABA, reducing the level of GABA needed to induce human islet cell proliferation.

## Discussion

GABA-R activation can protect ß-cells from apoptosis and promote their replication^2–6^. Oral GABA treatment increases human ß-cell replication several fold, typically from less than 1% to about 2–3% of adult human ß-cells in islet xenografts^4, 6^, which is similar to the maximum level of ß-cell replication that takes place shortly after birth. The ability of GABA treatment to promote ß-cell replication did not attenuate over the course of five weeks and led to increased ß-cell mass and function in human islet xenografts^6^. Additionally, long-term GABA treatment promotes the transdifferentiation of α-cells into ß-cells^7^. Accordingly, repurposing clinically applicable GABA_A_-R PAMs to enhance the activity of GABA secreted by ß-cells, or the effect of exogenous GABA, is an attractive new strategy for helping to treat diabetes. Moreover, combination treatment with GABA and a GABA_A_-R PAM may simultaneously inhibit pathogenic autoimmune responses that contribute to ß-cell destruction in T1D, as discussed below.

We first found that INS-1 cells express GABA_A_-R subunits that should confer benzodiazepine sensitivity and that these cells could produce low levels of GABA. Next, we asked whether three clinically applicable BBB-permeable benzodiazepine PAMs, as well as a peripherally restricted non-benzodiazepine PAM, could promote INS-1 cell proliferation. We observed that two of the four tested PAMs had a significant, albeit low, ability to promote INS-1 replication at least one of the dosages tested. This proliferative effect was independent of TSPO.

The PAM’s ability to increase INS-1 proliferation is likely to be mediated by enhancing the effect of endogenous GABA on activation of GABA_A_-Rs in an autocrine manner. Furthermore, while treatment with a low dose of GABA failed to stimulate significantly INS-1 cell proliferation, all of the tested PAMs at a nanomolar level enabled low doses of exogenous GABA to induce INS-1 cell proliferation *in vitro*.

Of the four GABA_A_-R PAMs that we tested on INS-1 cells, we carried alprazolam’s forward to study its effects on human islet cell replication because of alprazolam’s safety profile and a previous observation that alprazolam treatment reduced HbA1c levels in diabetics^15^, as discussed below. We observed that alprazolam (alone) enhanced human islet cell replication *in vitro*, most likely because it acted in conjunction with GABA secreted from ß-cells. Indeed, blocking GABA binding to GABA_A_-Rs with the antagonist bicuculline abrogated the ability of alprazolam to enhance islet cell replication. This suggests that ß-cell secreted GABA is involved in ß-cell replication in an autocrine manner and that this activity can be enhanced by a GABA_A_-R PAM. Addition of nifedipine to these cultures blocked alprazolam’s pro-mitotic effects, suggesting it enhances GABA_A_-R-mediated activation of the PI3K/Akt pathway^3^.

In combination, alprazolam and exogenous GABA had a greater ability to promote human islet-cell replication, and achieved a level of proliferation similar to that of GABA (alone) at ten-fold higher levels. The proliferating islet cells are likely to be primarily ß-cells because; 1) GABA increases human ß-cell, but not α-cell mass^6, 7^, 2) long-term GABA treatment is needed to promote the transdifferentiation of α-cells to ß-cells^7^ and such transdifferentiation would not require new DNA synthesis and would therefore not contribute to ^3^H-thymidine incorporation in our assays, and 3) islet δ and PP cells are not known to express GABA-Rs. While the spontaneous proliferation of non-ß-cells may have contributed in small part to the proliferation index of islet cells in all the *in vitro* cultures, our experiments with INS-1 cells provide direct evidence of the ability of GABA_A_-R PAMs to enhance endogenous GABA-stimulated ß-cell replication. We are currently conducting *in vivo* experiments to extend our *in vitro* observations.

A clinical trial conducted in the early 1990’s asked whether alprazolam treatment could help individuals who suffered from anxiety and also had poorly controlled T1D or T2D to better manage their diabetes. A group of nonanxious individuals with T1D or T2D who had poorly controlled blood glucose levels were also included in the study. Surprisingly, treatment with alprazolam, but not placebo, reduced blood HbA1c levels regardless of whether the patients suffered from anxiety^15^. These results were thought to stem from alprazolam’s modulation of neurotransmitter and neurohormone release. In light of our findings, it is possible that alprazolam’s beneficial effect on HbA1c may have arisen, at least in part, from enhancing the ability of islet GABA to promote ß-cell survival, replication and/or the transdifferentiation of α-cells into ß-cells.

We envision several different paths by which our findings could lead to clinical benefits. First, it may be possible to improve ß-cell mass and function in diabetics using BBB-permeable GABA_A_-R PAMs at doses below those used for CNS indications. Second, because the amount of ß-cell mass following T1D onset is a major factor determining the success of interventive therapy, a short-term PAM treatment may help preserve residual ß-cell mass and thereby improve the efficacy of interventive therapies. Along this line, GABA treatment enhanced ß-cell replication and survival in newly diabetic NOD mice and led to reversal of hyperglycemia^3, 7, 17^, indicating that enhancing GABA-R activity can be beneficial even when little ß-cell mass remains and there is robust ß-cell autoreactivity. Those observations are likely to reflect GABA’s ability to promote ß-cell replication/survival and α-cell transdifferentiation, as well as to inhibit inflammation (see below). Third, a short-term PAM treatment may help reduce ß-cell loss due to hypoxia and stress following islet transplantation, as suggested by the ability of GABA treatment to improve ß-cell survival in human islet xenografts^4, 6^. Finally, immune cells also express GABA-Rs and their activation can inhibit proinflammatory Th1, CD8^+^ and antigen-presenting cell responses^3, 5, 17–22^, as well as promote CD4^+^ Tregs^3, 23^. GABA-R activation has been shown to ameliorate disease in mouse models of T1D, experimental autoimmune encephalomyelitis, rheumatoid arthritis and T2D^3, 5, 17–24^. Therefore, GABA released from islet ß-cells may locally down-regulate inflammatory islet-infiltrates, and that propensity may be potentiated by treatment with a GABA_A_-R PAM. Furthermore, because the levels of circulating GABA may be insufficient to effectively inhibit inflammatory cells in the periphery, combination treatment with low doses of GABA and a GABA_A_-R PAM may suppress peripheral pro-inflammatory responses. Together, our observations suggest that GABA_A_-R PAMs may provide new avenues for safely enhancing ß-cell mass/function and simultaneously helping to control inflammatory immune responses that contribute to T1D and T2D pathogenesis.

## Methods

### Chemicals

Alprazolam, midazolam, clonazepam, PK11195, and bicuculline were purchased from Sigma-Aldrich. AP-3 was provided by Algiax Pharmaceuticals GmbH. Stock solutions of alprazolam (10 mM in DMSO), midazolam (6.25 mM in water), clonazepam (50 mM in ETOH), or AP-3 (50 mM in DMSO) were diluted into media to the indicated concentration.

### GAD enzymatic activity assay

INS-1 cells and 293 T cells (harvested in growth phase), as well as fresh mouse brain and human islets were stored at −80 C until use. The samples were rapidly homogenized into GAD activity assay buffer with protease inhibitors. The GAD enzymatic activity within homogenates was assessed using a standard CO^2^ trapping assay (in quadruplicate) as previously described^25^.

### Identification of GABA_A_-R subunits in INS-1 cells using qRT-PCR

We designed GABA_A_-R subunit-specific primers that spanned introns in genomic DNA. Total RNA was extracted from INS-1 cells using Qiagen’s RNeasy Mini Kit and was reversed transcribed into cDNA using ThermoFisher Scientific’s High-Capacity RNA-to-cDNA Kit. The presence of different GABA_A_-R subunit transcripts was assessed using qRT-PCR and the Applied Biosystem’s PowerUp SYBR Green Master Mix.

### Proliferation assays

INS-1 cell ^3^H-thymidine incorporation assay: INS-1 cells at 1 × 10^5^/well were treated in triplicate with indicated concentrations of individual compounds and cultured in 10% FCS RPMI164 medium in the presence of ^3^H-thymidine (0.3 μCi/well) for 48 hours (an optimal time period). The cells were harvested and the levels of ^3^H-thymidine uptake in individual wells were measured by a scintillation counter.

Islet cell proliferation assay: Fresh human islets were obtained from the Integrated Islet Distribution Program and islets (50–75 IEQ/well) were treated in triplicate with the indicated dosage of GABA, together with, or without, the indicated PAM in CMRL medium (0.1% glucose, Gibco) containing 10% human AB-type sera (MP Biomedicals, Santa Ana, USA) for 4 days in the presence of ^3^H thymidine (0.2 mCi/well) and then harvested and counted as above.

MTT (3-(4,5-dimethylthiazol-2-yl)-2,5-diphenyltetrazolium) assay: INS-1 cells at 1 × 10^5^/well were treated in quadruplicate with the indicated compounds in 10% FCS RPMI164 medium (phenol–free) for 44 hours. The cells were exposed to 20 µl of MTT (20 mg/ml, Sigma) for 4 hours. After the supernatants of cultured cells were removed, the resulting formazan in individual wells were dissolved in 100 ml DMSO and measured at absorbance of 570/650 nm in a microplate reader.

## Electronic supplementary material

Supplementary Fig. 1
