# Supplementary material for: Clinically applicable GABA receptor positive allosteric modulators promote ß-cell replication
Source: Sci Rep. 2017 Mar 23;7:374. doi: 10.1038/s41598-017-00515-y (PMC5428733; doi:10.1038/s41598-017-00515-y)
Supplement: Supplementary file 1 — Supplementary Fig. 1 [file 41598_2017_515_MOESM1_ESM.pdf]

Supplementary information

Clinically applicable GABA receptor positive allosteric modulators promote ß-cell replication

Jide Tian, Hoa Dang, Blake Middleton and Daniel L. Kaufman

Department of Molecular and Medical Pharmacology, University of California, Los Angeles, California

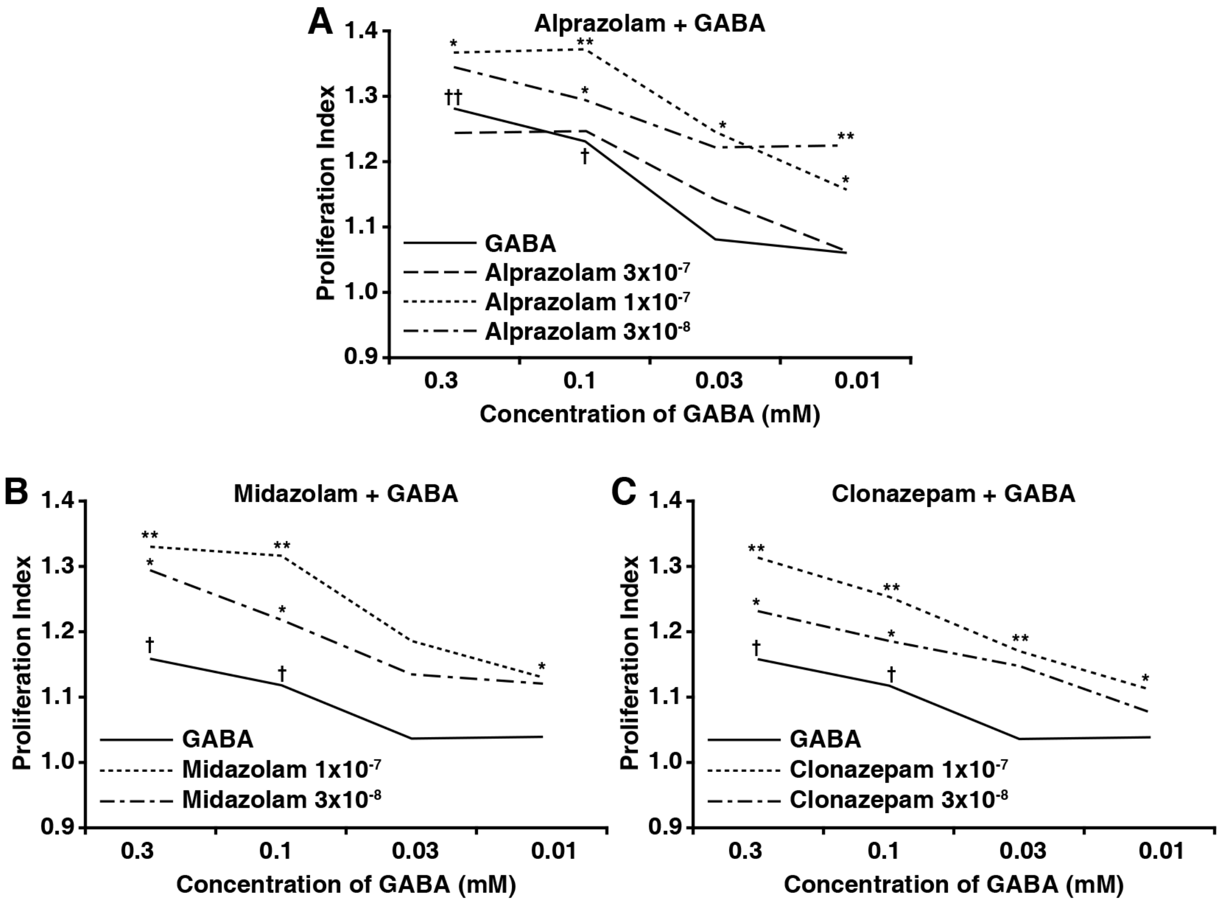

Supplementary Figure 1 legend: PAMs enhance GABA's ability to promote ß-cell replication as measured by MTT assay. INS-1 cells were treated with a dose range of GABA in the presence or absence of the indicated concentrations of B) alprazolam, C) midazolam, or D) clonazepam for 48 h. Control cells were incubated with medium alone. The cell proliferation was measured by MTT assay. Data shown are the average rate of proliferation relative to that of the control (designated as 1) from two separate experiments. $^{\dagger}p<0.05$, $^{\dagger\dagger}p<0.01$ versus control cultures with medium alone; $^{*}p<0.05$, $^{**}p<0.01$ versus cultures with the same dose of GABA, as determined by Student T test.
